# Very Late Recurrence in Breast Cancer: Is Breast Cancer a Chronic Disease?

**DOI:** 10.7759/cureus.22804

**Published:** 2022-03-03

**Authors:** Arimichi Kamata, Koji Hino, Koki Kamiyama, Yoshihiro Takasaka

**Affiliations:** 1 Surgery, Tomei Atsugi Hospital, Atsugi, JPN

**Keywords:** relapse-free interval, er-positive, chronic disease, late recurrence, breast cancer

## Abstract

Although breast cancer treatments have made great strides in recent decades, there are still many recurrences. Late recurrence is one of the characteristics of breast cancer. Here, we present four cases of recurrence more than 10 years after the initial diagnosis. The time from diagnosis to recurrence was 13 to 20 years in our four cases, which were all estrogen receptor (ER)-positive, and one was also human epidermal growth factor receptor 2-positive. Long-term hormone therapy for 10 years is necessary to prevent late recurrence of breast cancer, but we need to know that late recurrence remains common. Risk factors for late recurrence include ER positivity, progesterone receptor positivity, and low Ki67. The most common sites of recurrence are the lungs/pleura and bones, which was also the case in our experience. It is no exaggeration to say that breast cancer is a chronic disease similar to hypertension and diabetes. This is because breast cancer is not completely cured by surgery alone and lasts for a long time, with patients requiring long-term hormone therapy. Moreover, it can recur even after 10 years or more.

## Introduction

Drug therapy after breast cancer surgery has made great strides in recent decades. Postoperative tamoxifen for hormone receptor-positive breast cancer reduced the risk of recurrence by 40% [1]. Anthracycline and taxane anticancer drugs for breast cancer requiring chemotherapy such as triple-negative breast cancer also greatly reduced the risk of recurrence [2]. The achievements of perioperative trastuzumab and pertuzumab for human epidermal growth factor receptor 2 (HER2)-positive breast cancer are also noteworthy [3,4]. Advances in drug therapy have reduced postoperative recurrences and saved lives.

However, recurrence has not been completely suppressed. According to a large population study in the Netherlands [5], the 10-year postoperative recurrence-free survival rate was 87.5% in the luminal-A-type patient population. The recurrence-free survival rates in HER2-positive and triple-negative patient populations were 70.0% and 73.4%, respectively. Despite advances in drug treatment, many patients suffer from breast cancer recurrence.

Late recurrence is known to be one of the hallmarks of breast cancer. Similar to gastric cancer, colorectal cancer, and lung cancer, breast cancer usually recurs within five years after surgery; however, cases of recurrence are also reported five to ten years after surgery. Because the outpatient follow-up is completed about 10 years after the operation, recurrence after 10 years after the operation remains unknown. We encountered four cases of recurrence more than 10 years after the operation. Here, we present these cases and provide a brief review of the relevant literature.

These cases and discussions were previously presented as a poster during the 83rd Annual Congress of Japan Surgical Association on November 18-20, 2021.

## Case presentation

As of November 2021, there were four breast cancer patients in our hospital who had relapsed more than 10 years after the initial diagnosis. The characteristics of the four cases are summarized in Table 1. The age at the time of breast cancer diagnosis was 33 to 64 years. The relapse-free interval was 13 to 20 years. The details of each case are shown in Table 1.

**Table 1:** Patient characteristics. Bt: total mastectomy; Bp: partial mastectomy; Ax: axillary lymph node dissection; ER: estrogen receptor; PgR: progesterone receptor; HER2: human epidermal growth factor receptor 2

Patients	Age at diagnosis	Surgery	Pathological diagnosis of surgery	Postoperative adjuvant drug therapy	Relapse-free interval (years)	Site of relapse
Case 1	60	Bp + Ax	Invasive ductal carcinoma pT1a (3 mm + DCIS), pN0 (0/11) ER+, PgR+, HER2-	Anastrozole for 5 years	13	Lung, pleura, lymph node
Case 2	64	Bp + Ax	Invasive ductal carcinoma pT1c (19 mm), pN0(0/12) ER+, PgR-, HER2-	Letrozole for less than 1 year	14	Liver, lung, pleura, bone
Case 3	53	Bt + Ax	Invasive ductal carcinoma pN1 (1/8) ER+, PgR+, HER2-	CMF for 1 course, tamoxifen several months	20	Lung, bone, lymph node
Case 4	33	Bt + Ax	Invasive ductal carcinoma pN1 (2/22) ER+, PgR+, HER2+	UFT for 4 years, tamoxifen and goserelin for 5 years	16	Liver, lung, pleura

Case 1

A 60-year-old woman underwent a partial mastectomy and axillary lymph node dissection for left breast cancer in 2007. The pathological diagnosis was invasive ductal carcinoma, pT1a (3 mm + DCIS), pN0 (0/11), estrogen receptor (ER)-positive, progesterone receptor (PgR)-positive, and HER2-negative on immunostaining. After the surgery, she received 50 Gy of radiation therapy for her preserved breasts. She took anastrozole for five years. She was followed up for 11 years in a specialized outpatient department of breast surgery and ended her regular visits in 2018 due to no recurrence.

**Figure 1:**
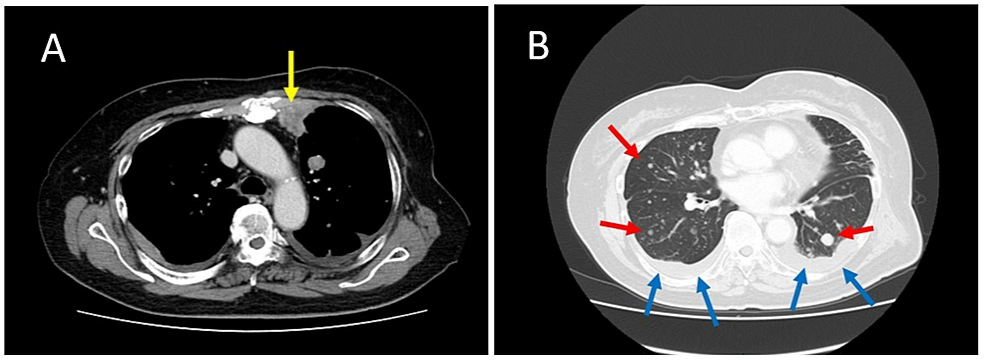
CT scan images. (A) The yellow arrow indicates the internal thoracic lymph node metastasis. (B) The red arrows indicate multiple lung metastases, and the blue arrows indicate pleural effusion. CT: computed tomography

In 2020, she underwent a preoperative chest X-ray when she broke her right arm which was notable for pleural effusion and lung tumors. Her chest computed tomography (CT) showed multiple tumors of both lungs, pleural effusion, and right internal thoracic lymphadenopathy (Figure 1). Initially diagnosed as a cancer of unknown primary, she underwent a thoracoscopic left lung tumor biopsy for her diagnosis. The pathological diagnosis was consistent with the metastasis of breast cancer; the tumor was ER-positive, PgR-positive, HER2 was 2+, and Ki67 was 10% on immunostaining. The fluorescence in situ hybridization (FISH) method for HER2 was negative. The HER2 score for immunostaining was different from 13 years ago and was possibly influenced by the heterogeneity of the tumor. We considered this tumor to be a recurrence of the tumor 13 years ago. She visited our breast surgery department again and started her treatment. She started treatment with chemotherapy because her metastases were a life-threatening condition. She received paclitaxel plus bevacizumab. The three months of treatment worked well, and her lung metastases and pleural effusions almost disappeared. Subsequent treatment was switched to hormone therapy. She started taking anastrozole and has continued without cancer progression for more than 11 months.

Case 2

A 64-year-old woman underwent a partial mastectomy and axillary lymph node dissection for right breast cancer in 2006. The pathological diagnosis was invasive ductal carcinoma, pT1c (19 mm), pN0 (0/12), ER-positive, PgR-negative, and HER2-negative by immunostaining. After the surgery, she received 50 Gy of radiation therapy on her preserved breasts. She started taking letrozole, but it was stopped after approximately a year due to an adverse event. She was followed up in the Breast Surgery Department until 2016. She had no recurrence at the end of the follow-up.

**Figure 2:**
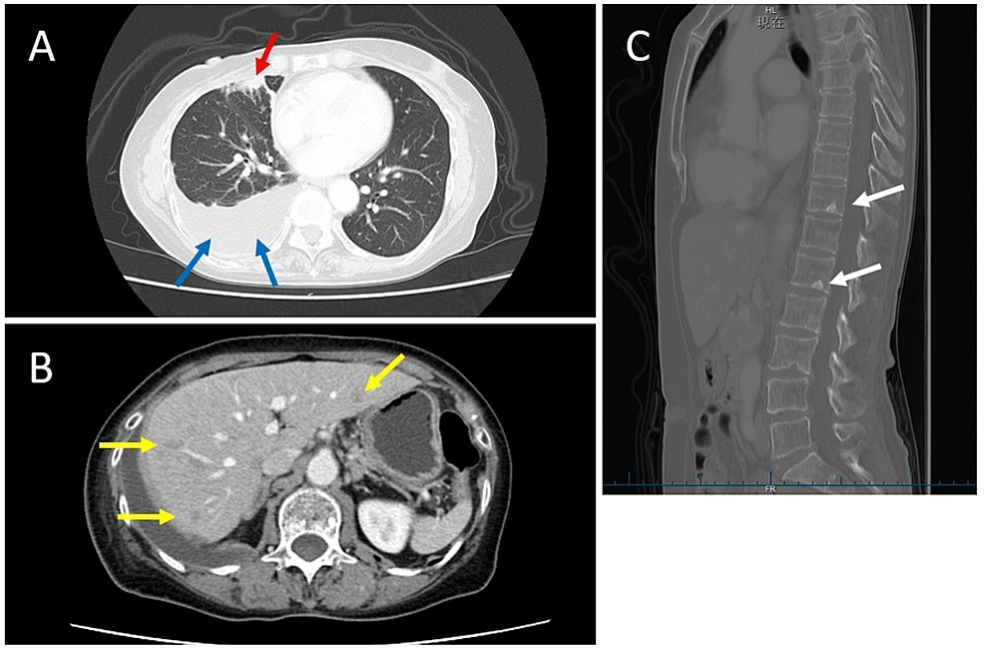
CT scan images. (A) The red arrow indicates lung metastasis, and the blue arrows indicate pleural effusion. (B) Yellow arrows indicate multiple liver metastases. (C) The white arrows indicate multiple bone metastases. CT: computed tomography

In 2020, she was pointed out for pleural effusion by an X-ray at her family doctor and was further examined at our hospital. Her CT scan confirmed multiple liver metastases, lung metastases, right pleural effusion, and bone metastases (Figure 2). She had elevated breast cancer tumor markers cancer antigen 15-3 (CA15-3) and NCC-ST439 and was diagnosed with a recurrence of breast cancer. There were no other primary lesions. She started treatment with chemotherapy because it was a life-threatening metastasis. She was 78-year-old at this point and was given eribulin, which has relatively few side effects. She received eribulin for five months, and her life-threatening metastases improved. She switched to hormone therapy with fewer side effects. She started taking anastrozole and has continued for more than five months without cancer progression.

Case 3

In 1999, a 53-year-old woman underwent a total mastectomy and axillary lymph node dissection for left breast cancer. Her pathological diagnosis was invasive ductal carcinoma, pN1 (1/8), ER-positive, PgR-positive, and HER2-negative on immunostaining. We could not get further information such as tumor diameter because the paper medical record was discarded. She received CMF therapy (cyclophosphamide, methotrexate, and fluorouracil) after her surgery, but only one course due to an adverse event. Oral tamoxifen was also discontinued in a few months due to an adverse event. She was followed up in the Breast Surgery Department until 2009. She had no recurrence at the end of the follow-up.

**Figure 3:**
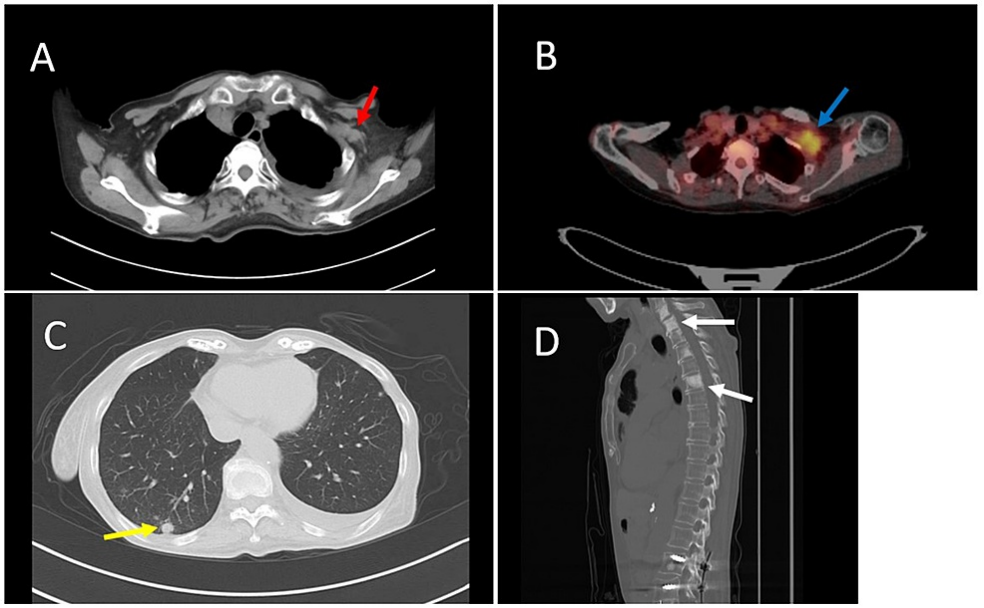
CT and PET-CT. (A, B) The red and blue arrows indicate lymph node metastases. (C) The yellow arrow indicates lung metastasis. (D) The white arrows indicate multiple bone metastases. CT: computed tomography; PET-CT: positron emission tomography-computed tomography

In 2019, she noticed a swelling of the left supraclavicular lymph node which was examined in our department. She was diagnosed with multiple lung metastases, multiple bone metastases, and supraclavicular lymph node metastases on positron emission tomography-computed tomography and contrast-enhanced CT (Figure 3). She was considered a case of breast cancer recurrence because of her elevated tumor marker CA15-3. We recommended hormone therapy because she did not have life-threatening metastases. She started taking anastrozole. However, similar to tamoxifen, she suffered from adverse events with anastrozole and stopped taking her medication in two months. Because she had been untreated for more than a year, a CT scan in 2021 showed that her tumor had worsened. Hence, we suggested taking capecitabine and she agreed. She has been taking capecitabine for over a year without any major adverse events. Her treatment has been effective without any cancer progression.

Case 4

In 2001, a 33-year-old woman underwent a total mastectomy and axillary lymph node dissection for right breast cancer. Her pathological diagnosis was invasive ductal carcinoma, pN1 (2/22), ER-positive, PgR-positive, and HER2-positive on immunostaining. ki67 was 10%. In this case, the paper chart was discarded, so we could not obtain information such as the tumor diameter. She took the anticancer drug UFT (tegafur and uracil) for four years after her surgery. She was also treated with tamoxifen and goserelin as hormone therapy for five years. The anti-HER2 drug trastuzumab was not used because as of 2001 there were no data showing its effectiveness as postoperative adjuvant treatment. Until 2011, she was followed up in the Breast Surgery Department. She ended her visit because she had no recurrence.

**Figure 4:**
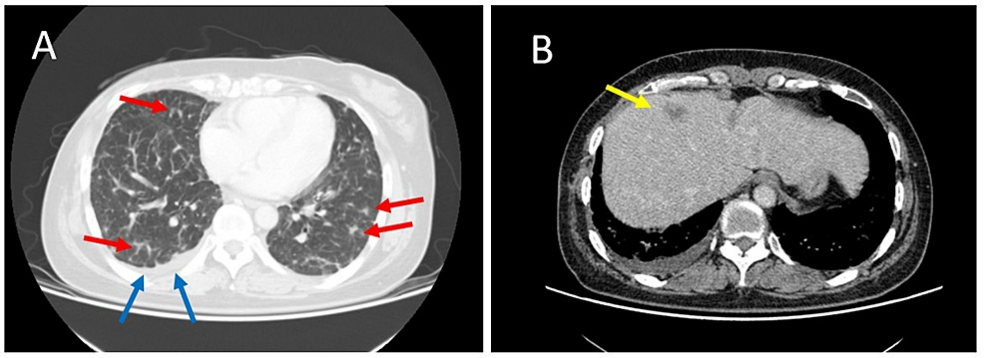
CT scan images. (A) The red arrows indicate multiple lung metastases, and the blue arrows indicate pleural effusion. (B) The yellow arrow indicates liver metastasis. CT: computed tomography

In 2017, she experienced respiratory distress and underwent various tests at another hospital. Her CT scan showed bilateral lung tumors, pleural effusion, and multiple liver tumors (Figure 4). Her condition was considered a cancer of unknown primary, and she underwent her thoracoscopic left upper lobectomy for her pathological diagnosis. The pathological diagnosis of her lung tumor was consistent with the metastasis of breast cancer. The tumor was ER-positive, PgR-positive, and HER2-positive on immunostaining. It was the same type as the first surgery. She visited our Breast Surgery Department again and started her treatment. Her metastases were life-threatening and she received docetaxel, trastuzumab, and pertuzumab as her first-line treatment. This treatment had a great effect. However, due to the adverse event of docetaxel, it was terminated after six courses. Similar to the Cleopatra trial policy [6], she began to receive trastuzumab and pertuzumab treatment. While continuing with these two drugs, she has maintained her tumor shrinkage for over four years.

## Discussion

Although the late recurrence of breast cancer remains unclear, it is closely related to tumor dormancy. Tumor dormancy describes a prolonged quiescent state in which tumor cells are present, but disease progression is not yet clinically apparent. Two different hypotheses are currently discussed: tumor cells persist either by completely withdrawing from the cell cycle or by continuing to proliferate at a slow rate that is counterbalanced by cell death [7].

We encountered four cases of late recurrence. The period from the first surgery to the confirmation of recurrence was 13 to 20 years. There are several case reports of late recurrence. Surprisingly, there are reports of recurrence more than 40 years after surgery [8]. There are various reports on the frequency of late recurrence of breast cancer. In the ATLAS trial [9], patients who took tamoxifen orally and had no recurrence five years after the surgery were enrolled. Of these, 13.1% relapsed between five and ten years after the surgery. Overall, 8.3% relapsed between 10 and 15 years after the surgery. This report of late recurrence frequency is higher than we expected, and we need to reaffirm late recurrence. In addition, it has recently been shown that long-term hormone therapy for 10 years after surgery decreases late recurrence after 10 years [9]. Of the four patients in this study, two had five years of hormone therapy and two had less than one year of hormone therapy. In these cases, if they had received 10 years of hormone therapy, recurrence could have been prevented.

All four cases we experienced were ER-positive. The risk of breast cancer recurrence is higher for ER-negative cases for up to three years after surgery. It reverses three years after the surgery, and ER-positive becomes higher after 3 years [10]. Another analysis of 10-year recurrence-free patients showed that ER-positive, PgR-positive, and low Ki67 were risk factors for late recurrence. This is in contrast to the results of the follow-up group for less than 10 years [11]. Regarding the site of recurrence, it is reported that it is more common in the lung, pleura, and bone and has fewer liver and brain metastases in groups over 10 years [11]. All four cases we presented also had lung metastases. Pleural effusion was observed in three cases. Metastatic sites tended to be the same as in this report.

It is no exaggeration to say that breast cancer, especially ER-positive breast cancer, is a chronic disease just like hypertension and diabetes. This is because breast cancer is not completely cured by surgery alone and lasts for a long time, patients require long-term hormone therapy, and it can recur even after 10 years or more. The term “chronic” comes from the Greek word khronikos or time. In case of illness, it means “persisting for a long time or constantly recurring” [12], which is consistent with the characteristics of breast cancer. Medical models such as the Cancer Survivorship Care Model manage breast cancer as a chronic disease and contribute to improving the long-term health of breast cancer survivors [13]. We need to explain to patients that breast cancer is a chronic disease and recommend long-term hormone therapy to prevent late recurrences. This is the same as recommending drug therapy for hypertension and diabetes to prevent cardiovascular events.

However, there are two major differences between breast cancer and hypertension or diabetes. The first is outpatient follow-up. Tracking for the possibility of recurrence after breast cancer surgery does not improve overall survival, and excessive testing only creates worry and cost problems [14,15]. As long as there are no symptoms, there is no need to follow up for longer than 10 years. Second, breast cancer is very different from other diseases such as hypertension and diabetes in terms of the mortality of distant recurrence. Doctors and patients may understand the term chronic disease differently [16]. The term must be used sparingly to arouse false hopes and avoid misleading.

## Conclusions

We encountered four cases of recurrence more than 10 years after the initial diagnosis. The frequency of late recurrence is high, and we need to be careful. Risk factors for late recurrence include ER positivity, PgR positivity, and low Ki67. The sites of late recurrence were more common in the lungs/pleura and bone. Hormonal therapy for 10 years is necessary to prevent late recurrence. Breast cancer, especially ER-positive breast cancer, requires long-term medication and can recur more than 10 years after surgery. Therefore, it is no exaggeration to say that this disease is a chronic disease.
